# Comparative Assessment of Forensic Odontology Awareness Among Dental and Medical Students in Turkey

**DOI:** 10.1111/eje.70148

**Published:** 2026-03-28

**Authors:** Emine Nur Kahraman, Büşra Yılmaz

**Affiliations:** ^1^ Department of Dentomaxillofacial Radiology, Faculty of Dentistry Istinye University Istanbul Turkey; ^2^ Department of Radiation Oncology The University of Texas MD Anderson Cancer center Houston Texas USA

**Keywords:** dental education, disaster victim identification, forensic odontology, interdisciplinary training, medical education

## Abstract

**Introduction:**

This study aimed to assess and compare the awareness and knowledge of forensic odontology among dental and medical students in Turkey with a focus on identifying educational gaps and evaluating interdisciplinary readiness.

**Materials and Methods:**

A cross‐sectional web‐based survey was conducted with 234 students (144 dental and 90 medical students) in their clinical years. The questionnaire covered five domains: demographics, exposure to forensic education, conceptual familiarity, terminology recognition and professional responsibilities. Associations between survey responses and demographic variables were analysed using Pearson's chi‐square test and Cramér's V.

**Results:**

Dental students reported higher frequencies of forensic exposure, with 67.4% completing coursework and 86.8% attending workshops, compared to 28.9% and 50.0% of medical students, respectively. Conversely, medical students demonstrated higher recognition rates in specific domains, such as lip print terminology (24.4% vs. 4.9%) and the utility of teeth as a DNA source (33.3% vs. 12.5%). Senior students and female participants exhibited significantly higher indices of awareness and ethical sensitivity. Notably, a marked dissonance was observed between participants' high self‐rated sufficiency (> 80%) and their limited technical familiarity with specific forensic parameters.

**Conclusions:**

Despite having greater curricular exposure, dental students did not consistently demonstrate superior forensic knowledge compared to medical students, highlighting a disconnect between educational quantity and professional proficiency. Widespread deficits in critical medicolegal areas suggest that current instructional models are insufficient.

## 1 Introduction

Forensic odontology (FO) is a cornerstone of human identification, particularly in disaster‐prone regions. In Turkey, which experiences over 35 000 annual earthquakes [1], the 2023 seismic events resulted in over 50 000 fatalities [2]. These mass casualty incidents necessitate robust identification protocols. Conventional methods, such as dactyloscopy, are frequently unfeasible due to post‐mortem degradation. Consequently, dental tissues represent a superior, resilient and cost‐effective identification substrate [3].

Historically, dental identification was the primary method for disaster victim identification (DVI) prior to the widespread adoption of DNA profiling [4]. However, in low‐ and lower‐middle‐income countries (LMICs), resource constraints often render exclusive reliance on DNA testing impractical [5]. In these settings, dental evidence remains critical, provided ante‐mortem records are accessible and interpretable. Success in DVI demands interdisciplinary cohesion; when medical professionals understand dental data limitations and dentists possess forensic competency, identification efficiency increases significantly.

Despite its strategic importance, FO remains marginalized in healthcare education. As a discipline bridging medicine, law and ethics [6, 7], its current academic positioning is inadequate. Recent global data highlight severe pedagogical gaps. In India, while 94% of students value dental records, fewer than 18% understand legal retention protocols or age estimation methods [8]. Similar educational deficits exist in Saudi Arabia [9], Mumbai [10], Egypt [11] and Afghanistan [12], where curricula lack structured practical training.

In Turkey, the situation mirrors these global trends. Although 92.9% of dentists support FO integration in DVI, only 20.5% are familiar with current identification protocols, and nearly half of dental students lack formal forensic education [13]. This deficit is compounded by a global shortage of trained forensic odontologists [5]. Furthermore, medical students represent key stakeholders in medico‐legal investigations but rarely encounter dental forensic principles during clinical rotations. Effective participation in legal investigations requires targeted, practical training for both cohorts [14].

Integrating FO into undergraduate curricula enhances recordkeeping accuracy and fosters necessary interdisciplinary collaboration [8]. Early exposure promotes systematic documentation and instills ethical responsibility. As forensic service demand rises, equipping future providers with this specialized knowledge is urgent.

This study evaluated the educational exposure, self‐perceived sufficiency and objective understanding of forensic odontology among dental and medical students in Turkey. By assessing these cohorts simultaneously, we aimed to identify specific pedagogical patterns that hinder interdisciplinary collaboration in medicolegal investigations. Accordingly, the primary research question was to determine whether variations in curricular exposure correlate with measurable differences in forensic awareness. We hypothesized that dental students, having higher curricular exposure, would demonstrate greater conceptual and technical familiarity with forensic odontology than medical students. Furthermore, we anticipated that formal training history would be associated with awareness levels across both groups.

## 2 Materials and Methods

### 2.1 Study Design, Setting and Sampling

This cross‐sectional study was conducted in accordance with the Checklist for Reporting Results of Internet E‐Surveys (CHERRIES) [15]. The research setting comprised two National Accreditation Board‐certified universities in Istanbul offering fully English‐medium undergraduate programs in Medicine and Dentistry. The study population was stratified by clinical progression into ‘Junior’ (clinical induction phase: 4th‐year dental, 5th‐year medical students) and ‘Senior’ (pre‐graduation phase: 5th‐year dental, 6th‐year medical students). Data were collected between December 1 and December 14, 2024.

Participants were recruited via a non‐probability convenience sampling strategy. Survey links were disseminated through official institutional mailing lists and student representative communication channels. Participation was voluntary, and no incentives were offered.

Sample size was determined a priori based on the planned comparison of categorical variables. Given that the primary analyses relied on chi‐square tests, an a priori power analysis was conducted using G*Power 3.1. Assuming a medium effect size (Cohen's w = 0.30), α = 0.05, statistical power = 0.95 and 2 degrees of freedom, the minimum required sample size was calculated as 172 participants. Owing to the voluntary and non‐probability sampling design, subgroup sizes were not predefined or constrained.

### 2.2 Instrument Development

The data collection instrument was developed as an exploratory descriptive tool following AMEE (The International Association for Health Professions Education) guidelines [16]. Initial literature review was conducted to identify relevant conceptual domains and existing questionnaire items in forensic odontology education. Based on this review, two previously published questionnaire studies, one conducted among dental students and the other among medical students, were used as the primary sources for item adaptation [7, 17]. Validity was established through a multi‐stage protocol. First, content validity was assessed by certified oral and maxillofacial radiologists who independently evaluated the initial item pool for technical accuracy and relevance. Second, cognitive evaluation was performed through semi‐structured interviews with representative students to ensure conceptual clarity and verify that terminology was interpreted as intended. Finally, a pilot study was conducted with a stratified sample of 20 students (5 junior and 5 senior students from each faculty) to assess face validity and feasibility. The specific objectives of the pilot were to evaluate item clarity, response latency, platform functionality and overall feasibility. Based on pilot feedback, minor syntax adjustments were made to two items. Students who participated in the pilot phase were excluded from the main study sample to prevent priming bias. The final 25‐item instrument is provided in Supplementary File S1.

The final questionnaire comprised 25 items organized into two sections. The first section consisted of five demographic items addressing gender, age group, field of study and academic year (Q1–Q5). The second section included 20 content items (Q6–Q25) grouped into four conceptual domains: exposure to forensic education and sources of knowledge (Q6–Q8), conceptual familiarity with forensic odontology (Q9–Q18), terminology recognition (Q19–Q21) and self‐perception and professional responsibility (Q22–Q25). Most items employed dichotomous response options, while three items (Q3, Q14 and Q15) allowed multiple selections.

### 2.3 Data Collection and Integrity

The survey was hosted on Google Forms and administered in English. Given that the study population comprised students enrolled in English‐medium curricula with documented proficiency requirements (e.g., TOEFL, IELTS), linguistic translation and calibration were not required.

To balance data integrity with participant anonymity, the platform was configured to restrict submissions to a single entry per user. This was achieved by enabling the ‘Limit to 1 response’ setting while the ‘Collect email addresses’ feature remained disabled. While this configuration required a temporary login for validation purposes, it did not record user identities, email addresses, or IP identifiers in the final dataset. To minimize social desirability bias and address potential privacy concerns, the informed consent section clarified that this authentication step was technical in nature and intended to prevent duplicate entries.

### 2.4 Statistical Analysis

Statistical analyses were performed using IBM SPSS Statistics for Windows, Version 26.0 (IBM Corp., Armonk, NY, USA). Categorical variables were summarized as frequencies and percentages. Associations between survey responses and demographic variables (sex, age group, academic year) were assessed using Pearson's chi‐square test, with Fisher's exact test applied when expected cell counts were below five. Effect size was quantified using Cramér's V. Normality testing was not applicable. Statistical significance was set at a two‐tailed *p* < 0.05.

## 3 Results

A total of 234 students were included in the analysis, comprising 144 dental and 90 medical students. The majority of participants were female, aged 22–25 years, with a comparable distribution of junior and senior students across the two disciplines (Table 1).

**TABLE 1:** Demographic characteristics.

	Dental students [*n* (%)]	Medical students [*n* (%)]
Gender	Male: 46 (31.9%)	Male: 42 (46.7%)
	Female: 98 (68.1%)	Female: 48 (53.3%)
Age group	18–21: 25 (17.6%)	18–21: 11 (15.5%)
	22–25: 116 (81.7%)	22–25: 52 (73.2%)
	> 25: 1 (0.7%)	> 25: 8 (11.3%)
Year of study	Junior Year (4th Year): 90 (62.5%)	Junior Year (5th Year): 48 (53.3%)
	Senior Year (5th Year): 54 (37.5%)	Senior Year (6th Year): 39 (43.3%)

Overall exposure to forensic odontology differed markedly between dental and medical students. Dental students more frequently reported completion of formal forensic coursework (67% vs. 29%) and participation in workshops or related training activities (87% vs. 50%). Awareness of the general role of forensic odontology in the identification of deceased individuals was high in both groups; however, it was more prevalent among dental students (92% vs. 68%). In contrast, medical students more frequently recognized specific identification‐related applications, particularly the role of dental records (Table 2).

**TABLE 2:** Survey responses of dental and medical students regarding forensic odontology.

Survey questions	Dental students [*n* (%)]	Medical students [*n* (%)]
Have you completed any formal coursework specifically covering forensic odontology? (e.g., elective or core classes)	Yes: 97 (67.4%) No: 47 (32.6%)	Yes: 26(28.9%) No: 64 (71.1%)
2 Have you attended any workshops, seminars, or trainings focused on forensic odontology within the past 2 years?	Yes: 125 (86.8%) No: 19 (13.2%)	Yes: 45 (50%) No: 45 (50%)
3 What is the source of your knowledge about forensic dentistry?*	Books: 2 (1.4%) Internet: 54 (37.5%) Lectures: 20 (13.9%) ≥ 2 selections: 44 (30.6%) I don't know: 24 (16.7%)	Books: 10 (11.1%) Internet: 22 (24.4%) Lectures: 3 (3.3%) ≥ 2 selections: 46 (51.1%) I don't know: 9 (10.0%)
4 Are you familiar with the concept of forensic odontology as a branch of dentistry?	Yes: 39 (27.1%) No: 105 (72.9%)	Yes: 13 (14.4%) No: 77 (85.6%)
5 Are you familiar with the concept of forensic odontology as a branch of forensic medicine?	Yes: 31(21.5%) No: 113 (78.5%)	Yes: 35 (38.9%) No: 55 (61.1%)
6 Are you familiar with the concept that dentists may testify as expert witnesses in court for forensic cases?	Yes: 32 (22.2%) No: 112 (77.8%)	Yes: 37(41.1%) No: 53 (58.9%)
7 Are you familiar with the concept that maintaining accurate dental records contributes to quality patient care?	Yes: 20 (13.9%) No: 124 (86.1%)	Yes: 41 (45.6%) No: 49(54.4%)
8 Are you familiar with the concept that maintaining accurate dental records can assist in forensic identification?	Yes: 20(13.9%) No: 124(86.1%)	Yes: 41 (45.6%) No: 49(54.4%)
9 Are you familiar with the concept of estimating biological sex using teeth in forensic cases?	Yes: 30 (20.8%) No: 114 (79.2%)	Yes: 26 (28.9%) No: 64(71.1%)
10 Are you familiar with the concept of age estimation through forensic odontology?	Yes: 14 (9.7%) No: 130 (90.3%)	Yes: 39(43.3%) No: 51 (56.7%)
11 Are you familiar with the concept of using teeth as a source of DNA in forensic investigations?	Yes: 18(12.5%) No:126 (87.5%)	Yes: 30 (33.3%) No: 60(66.7%)
12 Are you familiar with the concept of bite mark analysis in forensic investigations?	Yes: 13(9.0%) No: 131 (91.0%)	Yes: 1(1.1%) No: 89 (98.9%)
13 Are you familiar with the concept that forensic odontology is used to help identify deceased individuals or crime suspects?	Yes: 132 (91.7%) No: 12 (8.3%)	Yes: 61 (67.8%) No: 29 (32.2%)
14 Which methods are used to determine the dental age of children and adults?*	Eruption patterns: 37 (25.7%) Histological methods: 2 (1.4%) Biochemical methods: 2 (1.4%) ≥ 2 selections: 92 (63.9%) I do not know: 11(7.6%)	Eruption patterns: 8 (8.9%) Histological methods: 3 (3.3%) Biochemical methods: 7 (7.8%) ≥ 2 selections: 62 (68.9%) I do not know: 10(11.1%)
15 Which techniques are used to determine the age and sex of a deceased person in mass disasters?*	Reconstruct body: 3 (2.1%) Dental records: 37 (25.7%) Fingerprints: 5 (3.5%) ≥ 2 selections: 85 (59.0%) I do not know: 14 (9.7%)	Reconstruct body: 7 (7.8%) Dental records: 17 (18.9%) Fingerprints: 3 (3.3%) ≥ 2 selections: 61 (67.8%) I do not know: 2 (2.2%)
16 What is the study of lip prints in forensic dentistry called?	Lipology: 88 (61.1%) Cheiloscopy: 7 (4.9%) Dermatoglyphics: 2 (1.4%) I do not know: 47 (32.6%)	Lipology: 16 (17.8%) Cheiloscopy: 22 (24.4%) Dermatoglyphics: 30 (33.3%) I do not know: 22 (23.4%)
17 Would you describe your knowledge and awareness of forensic odontology as sufficient?	Yes: 121 (84.0%) No: 23 (16.0%)	Yes: 74 (82.2%) No: 16 (17.8%)
18 Are you interested in participating in workshops and seminars in forensic odontology?	Yes: 45 (31.3%) No: 99 (68.8%)	Yes: 46 (51.1%) No: 44 (48.9%)
19 Which of the following signs may indicate child abuse?	Physical injuries: 9 (6.3%) Behavioural changes:13 (9.0%) Clothing: 0 (0.0%) Any scars: 2 (1.4%) All the above: 118 (81.9%) I do not know: 2 (1.4%)	Physical injuries: 3 (3.3%) Behavioural changes:17 (18.9%) Clothing: 13 (14.4%) Any scars: 16 (17.8%) All the above: 37 (41.1%) I do not know: 4 (4.4%)
20 What would you do if you suspected a child was being abused?	Inform police: 126 (87.5%) Inform parents: 18 (12.5%) Take no action: 0 (0.0%)	Inform police: 61 (67.8%) Inform parents: 20 (22.2%) Take no action: 9(10.0%)

*Note:* An asterisk (*) indicates that multiple responses were allowed for that item.

Discipline‐specific variation was also observed in relation to specialized forensic techniques. Familiarity with bite mark analysis was limited overall (9% vs. 1%), whereas awareness of teeth as a DNA source was higher among medical students (33% vs. 13%). Despite these gaps, most participants rated their overall knowledge as sufficient (84% and 82%, respectively). Nevertheless, interest in further training was more common among medical students (51% vs. 31%). In addition, dental students demonstrated greater recognition of child abuse indicators (82% vs. 41%) and a higher likelihood of reporting suspected cases (88% vs. 68%) (Table 2).

Within the dental student cohort, academic year showed significant associations with several knowledge‐related outcomes. Senior students were more likely to have completed forensic odontology coursework (*p* < 0.001) and demonstrated higher accuracy across key knowledge‐based items, including identification of knowledge sources, lip print terminology and dental age estimation. Gender was associated with identification awareness and dental age estimation knowledge (*p* < 0.01), whereas age group showed no consistent associations with the assessed outcomes (Tables 3 and 4).

**TABLE 3:** Correlations of dental students answers to survey questions with demographic characteristics.

Survey questions	Gender (v, *p*)	Age group (ν, *p*)	Junior/Senior Year (ν, *p*)
Have you completed any formal coursework specifically covering forensic odontology? (e.g., elective or core classes)	ν = 0.127, *p* = 0.129	ν = 0.150, *p* = 0.349	ν = 0.532, ***p* < 0.001**
2 Have you attended any workshops, seminars, or trainings focused on forensic odontology within the past 2 years?	ν = 0.173, ***p* = 0.038**	ν = 0.223, *p* = 0.129	ν = 0.048, *p* = 0.621
3 What is the source of your knowledge about forensic dentistry?*	ν = 0.247, *p* = 0.061	ν = 0.138, *p* = 0.667	ν = 0.519, ***p* < 0.001**
4 Are you familiar with the concept of forensic odontology as a branch of dentistry?	ν = 0.049, *p* = 0.558	ν = 0.191, *p* = 0.151	ν = 0.149, *p* = 0.084
5 Are you familiar with the concept of forensic odontology as a branch of forensic medicine?	ν = 0.033, *p* = 0.695	ν = 0.102, *p* = 0.619	ν = 0.231, ***p* = 0.006**
6 Are you familiar with the concept that dentists may testify as expert witnesses in court for forensic cases?	ν = 0.028, *p* = 0.738	ν = 0.129, *p* = 0.474	ν = 0.173, *p* = 0.061
7 Are you familiar with the concept that maintaining accurate dental records contributes to quality patient care?	ν = 0.109, *p* = 0.239	ν = 0.072, *p* = 0.658	ν = 0.013, *p* = 1.000
8 Are you familiar with the concept that maintaining accurate dental records can assist in forensic identification?	ν = 0.112, *p* = 0.177	ν = 0.063, *p* = 1.000	ν = 0.104, *p* = 0.320
9 Are you familiar with the concept of estimating biological sex using teeth in forensic cases?	ν = 0.089, *p* = 0.288	ν = 0.076, *p* = 1.000	ν = 0.009, *p* = 1.000
10 Are you familiar with the concept of age estimation through forensic odontology?	ν = 0.127, *p* = 0.127	ν = 0.056, *p* = 1.000	ν = 0.012, *p* = 1.000
11 Are you familiar with the concept of using teeth as a source of DNA in forensic investigations?	ν = 0.034, *p* = 0.685	ν = 0.071, *p* = 0.827	ν = 0.076, *p* = 0.442
12 Are you familiar with the concept of bite mark analysis in forensic investigations?	ν = 0.096, *p* = 0.249	ν = 0.050, *p* = 1.000	ν = 0.144, *p* = 0.132
13 Are you familiar with the concept that forensic odontology is used to help identify deceased individuals or crime suspects?	ν = 0.278, ***p* = 0.002**	ν = 0.196, *p* = 0.149	ν = 0.182, *p* = 0.057
14 Which methods are used to determine the dental age of children and adults?*	ν = 0.317, ***p* = 0.001**	ν = 0.141, *p* = 0.268	ν = 0.334, ***p* < 0.001**
15 Which techniques are used to determine the age and sex of a deceased person in mass disasters?*	ν = 0.147, *p* = 0.556	ν = 0.081, *p* = 0.978	ν = 0.192, *p* = 0.265
16 What is the study of lip prints in forensic dentistry called?	ν = 0.317, ***p* = 0.001**	ν = 0.141, *p* = 0.268	ν = 0.334, ***p* < 0.001**
17 Would you describe your knowledge and awareness of forensic odontology as sufficient?	ν = 0.108, *p* = 0.226	ν = 0.203, *p* = 0.180	ν = 0.250, ***p* = 0.004**
18 Are you interested in participating in workshops and seminars in forensic odontology?	ν = 0.052, *p* = 0.531	ν = 0.080, *p* = 0.945	ν = 0.027, *p* = 0.853
19 Which of the following signs may indicate child abuse?	ν = 0.223, *p* = 0.113	ν = 0.210, *p* = 0.129	ν = 0.254, ***p* = 0.035**
20 What would you do if you suspected a child was being abused?	ν = 0.079, *p* = 0.344	ν = 0.138, *p* = 0.394	ν = 0.033, *p* = 0.798

*Note:* Cramér's V (ν) values indicate the effect size of the associations between categorical variables. The questions marked with asterisks (*) indicate multiple responses. Statistical significance was set at *p* < 0.05.

**TABLE 4:** Chi‐square test results showing significant differences in dental students' responses by demographic characteristics.

Survey questions	Gender (*p*)	Age group (*p*)	Junior/Senior year (*p*)
Have you completed any formal coursework specifically covering forensic odontology? (e.g., elective or core classes)	*p* = 0.184	*p* = 0.443	***p* < 0.001**
2 Have you attended any workshops, seminars, or trainings focused on forensic odontology within the past 2 years?	*p* = 0.070	*p* = 0.169	*p* = 0.751
3 What is the source of your knowledge about forensic dentistry?*	*p* = 0.070	*p* = 0.542	***p* < 0.001**
4 Are you familiar with the concept of forensic odontology as a branch of dentistry?	*p* = 0.700	*p* = 0.169	*p* = 0.110
5 Are you familiar with the concept of forensic odontology as a branch of forensic medicine?	*p* = 0.861	*p* = 0.727	***p* = 0.010**
6 Are you familiar with the concept that dentists may testify as expert witnesses in court for forensic cases?	*p* = 0.905	*p* = 0.514	*p* = 0.062
7 Are you familiar with the concept that maintaining accurate dental records contributes to quality patient care?	*p* = 0.239	*p* = 1.000	*p* = 1.000
8 Are you familiar with the concept that maintaining accurate dental records can assist in forensic identification?	*p* = 0.275	*p* = 0.845	*p* = 0.320
9 Are you familiar with the concept of estimating biological sex using teeth in forensic cases?	*p* = 0.399	*p* = 1.000	*p* = 1.000
10 Are you familiar with the concept of age estimation through forensic odontology?	*p* = 0.141	*p* = 1.000	*p* = 1.000
11 Are you familiar with the concept of using teeth as a source of DNA in forensic investigations?	*p* = 0.893	*p* = 0.684	*p* = 0.515
12 Are you familiar with the concept of bite mark analysis in forensic investigations?	*p* = 0.349	*p* = 1.000	*p* = 0.132
13 Are you familiar with the concept that forensic odontology is used to help identify deceased individuals or crime suspects?	***p* = 0.003**	*p* = 0.149	***p* = 0.031**
14 Which methods are used to determine the dental age of children and adults?*	*p* = 0.933	*p* = 0.614	*p* = 0.829
15 Which techniques are used to determine the age and sex of a deceased person in mass disasters?*	*p* = 0.558	*p* = 0.956	*p* = 0.269
16 What is the study of lip prints in forensic dentistry called?	***p* = 0.003**	*p* = 0.251	***p* < 0.001**
17 Would you describe your knowledge and awareness of forensic odontology as sufficient?	*p* = 0.294	*p* = 0.225	***p* = 0.006**
18 Are you interested in participating in workshops and seminars in forensic odontology?	*p* = 0.664	*p* = 0.829	*p* = 0.889
19 Which of the following signs may indicate child abuse?	*p* = 0.113	*p* = 0.149	***p* = 0.026**
20 What would you do if you suspected a child was being abused?	*p* = 0.499	*p* = 0.394	*p* = 0.896

*Note:* Values represent *p*‐values derived from chi‐square tests. The questions marked with asterisks (*) indicate multiple responses. Statistical significance was set at *p* < 0.05.

Among medical students, both gender and academic year were significantly associated with multiple outcomes. Completion of forensic coursework and awareness of identification roles were more frequent among female and senior students (*p* < 0.001). Senior students also outperformed juniors on applied knowledge items, including age estimation and lip print terminology. Awareness of the importance of dental records and self‐reported knowledge sufficiency were significantly associated with gender, age group and academic year (Tables 5 and 6).

**TABLE 5:** Correlations of medical students answers to survey questions with demographic characteristics.

Survey questions	Gender (ν, *p*)	Age group (ν, *p*)	Junior/Senior Year (ν, *p*)
Have you completed any formal coursework specifically covering forensic odontology? (e.g., elective or core classes)	ν = 0.400, ***p* < 0.0001**	ν = 0.121, *p* = 0.349	ν = 0.339, ***p* < 0.001**
2 Have you attended any workshops, seminars, or trainings focused on forensic odontology within the past 2 years?	ν = 0.089, *p* = 0.398	ν = 0.084, *p* = 0.944	ν = 0.189, *p* = 0.265
3 What is the source of your knowledge about forensic dentistry?*	ν = 0.256, *p* = 0.216	ν = 0.230, *p* = 0.278	ν = 0.306, ***p* = 0.040**
4 Are you familiar with the concept of forensic odontology as a branch of dentistry?	ν = 0.004, *p* = 0.968	ν = 0.270, *p* = 0.079	ν = 0.141, *p* = 0.400
5 Are you familiar with the concept of forensic odontology as a branch of forensic medicine?	ν = 0.122, *p* = 0.248	ν = 0.089, *p* = 0.904	ν = 0.168, *p* = 0.300
6 Are you familiar with the concept that dentists may testify as expert witnesses in court for forensic cases?	ν = 0.033, *p* = 0.753	ν = 0.226, *p* = 0.211	ν = 0.168, *p* = 0.310
7 Are you familiar with the concept that maintaining accurate dental records contributes to quality patient care?	ν = 0.412, ***p* < 0.001**	ν = 0.301, ***p* = 0.041**	ν = 0.516, ***p* < 0.001**
8 Are you familiar with the concept that maintaining accurate dental records can assist in forensic identification?	ν = 0.173, *p* = 0.138	ν = 0.142, *p* = 0.644	ν = 0.181, *p* = 0.277
9 Are you familiar with the concept of estimating biological sex using teeth in forensic cases?	ν = 0.301, ***p* = 0.004**	ν = 0.188, *p* = 0.373	ν = 0.248, *p* = 0.060
10 Are you familiar with the concept of age estimation through forensic odontology?	ν = 0.099, *p* = 0.398	ν = 0.212, *p* = 0.273	ν = 0.263, ***p* = 0.036**
11 Are you familiar with the concept of using teeth as a source of DNA in forensic investigations?	ν = 0.047, *p* = 0.823	ν = 0.181, *p* = 0.407	ν = 0.256, *p* = 0.062
12 Are you familiar with the concept of bite mark analysis in forensic investigations?	ν = 0.099, *p* = 0.347	ν = 0.091, *p* = 1.000	ν = 0.144, *p* = 0.132
13 Are you familiar with the concept that forensic odontology is used to help identify deceased individuals or crime suspects?	ν = 0.597, ***p* < 0.001**	ν = 0.226, *p* = 0.210	ν = 0.597, ***p* < 0.001**
14 Which methods are used to determine the dental age of children and adults?*	ν = 0.387, *p* = 0.004	ν = 0.237, *p* = 0.221	ν = 0.697, ***p* = 0.001**
15 Which techniques are used to determine the age and sex of a deceased person in mass disasters?*	ν = 0.268, *p* = 0.153	ν = 0.200, *p* = 0.535	ν = 0.375, ***p* = 0.009**
16 What is the study of lip prints in forensic dentistry called?	ν = 0.155, *p* = 0.571	ν = 0.238, *p* = 0.080	ν = 0.359, ***p* < 0.001**
17 Would you describe your knowledge and awareness of forensic odontology as sufficient?	ν = 0.377, ***p* < 0.001**	ν = 0.223 *p* = 0.207	ν = 0.415, ***p* = 0.001**
18 Are you interested in participating in workshops and seminars in forensic odontology?	ν = 0.021, *p* = 0.844	ν = 0.176, *p* = 0.444	ν = 0.085, *p* = 0.692
19 Which of the following signs may indicate child abuse?	ν = 0.171, *p* = 0.775	ν = 0.226, *p* = 0.539	ν = 0.215, *p* = 0.590
20 What would you do if you suspected a child was being abused?	ν = 0.600, ***p* < 0.001**	ν = 0.148, *p* = 0.712	ν = 0.490, ***p* < 0.001**

*Note:* Cramér's V (ν) values represent the effect size of the associations between categorical variables. The questions marked with asterisks indicate multiple responses. Statistical significance was set at *p* < 0.05.

**TABLE 6:** Chi‐square test results showing significant differences in medical students' responses by demographic characteristics.

Survey questions	Gender (*p*)	Age group (*p*)	Junior/Senior year (*p*)
Have you completed any formal coursework specifically covering forensic odontology? (e.g., elective or core classes)	***p* < 0.001**	*p* = 0.687	***p* = 0.005**
2 Have you attended any workshops, seminars, or trainings focused on forensic odontology within the past 2 years?	*p* = 0.526	*p* = 0.921	*p* = 0.302
3 What is the source of your knowledge about forensic dentistry?*	*p* = 0.192	*p* = 0.300	***p* = 0.049**
4 Are you familiar with the concept of forensic odontology as a branch of dentistry?	*p* = 1.000	*p* = 0.062	*p* = 0.607
5 Are you familiar with the concept of forensic odontology as a branch of forensic medicine?	*p* = 0.348	*p* = 0.928	*p* = 0.328
6 Are you familiar with the concept that dentists may testify as expert witnesses in court for forensic cases?	*p* = 0.920	*p* = 0.198	*p* = 0.336
7 Are you familiar with the concept that maintaining accurate dental records contributes to quality patient care?	***p* < 0.001**	***p* = 0.050**	***p* < 0.001**
8 Are you familiar with the concept that maintaining accurate dental records can assist in forensic identification?	*p* = 0.153	*p* = 0.643	*p* = 0.315
9 Are you familiar with the concept of estimating biological sex using teeth in forensic cases?	*p* = 0.823	*p* = 0.397	*p* = 0.079
10 Are you familiar with the concept of age estimation through forensic odontology?	*p* = 0.398	*p* = 0.269	***p* = 0.036**
11 Are you familiar with the concept of using teeth as a source of DNA in forensic investigations?	*p* = 0.823	*p* = 0.397	*p* = 0.079
12 Are you familiar with the concept of bite mark analysis in forensic investigations?	*p* = 1.000	*p* = 1.000	*p* = 0.467
13 Are you familiar with the concept that forensic odontology is used to help identify deceased individuals or crime suspects?	***p* < 0.001**	*p* = 0.222	***p* < 0.001**
14 Which methods are used to determine the dental age of children and adults?*	***p* = 0.004**	*p* = 0.181	***p* < 0.001**
15 Which techniques are used to determine the age and sex of a deceased person in mass disasters?*	*p* = 0.155	*p* = 0.479	***p* = 0.001**
16 What is the study of lip prints in forensic dentistry called?	*p* = 0.557	*p* = 0.064	***p* = 0.003**
17 Would you describe your knowledge and awareness of forensic odontology as sufficient?	***p* < 0.001**	*p* = 0.166	***p* = 0.001**
18 Are you interested in participating in workshops and seminars in forensic odontology?	*p* = 1.000	*p* = 0.444	*p* = 0.692
19 Which of the following signs may indicate child abuse?	*p* = 0.781	*p* = 0.379	*p* = 0.712
20 What would you do if you suspected a child was being abused?	***p* < 0.001**	*p* = 0.542	***p* < 0.001**

*Note:* Values represent *p*‐values obtained from chi‐square tests. The questions marked with asterisks indicate multiple responses. Statistical significance was set at *p* < 0.05.

Analysis of individual knowledge‐based items revealed persistent discipline‐specific gaps. Correct responses for dental age estimation were provided by 31.3% of dental students and 44.4% of medical students (Figure 1A). Accuracy for age and sex identification techniques in mass disaster scenarios remained low in both groups (24.3% and 24.4%, respectively; Figure 1B). Knowledge of lip print terminology was particularly limited among dental students, with only 4.9% responding correctly, compared with 24.4% of medical students (Figure 1C).

**FIGURE 1:**
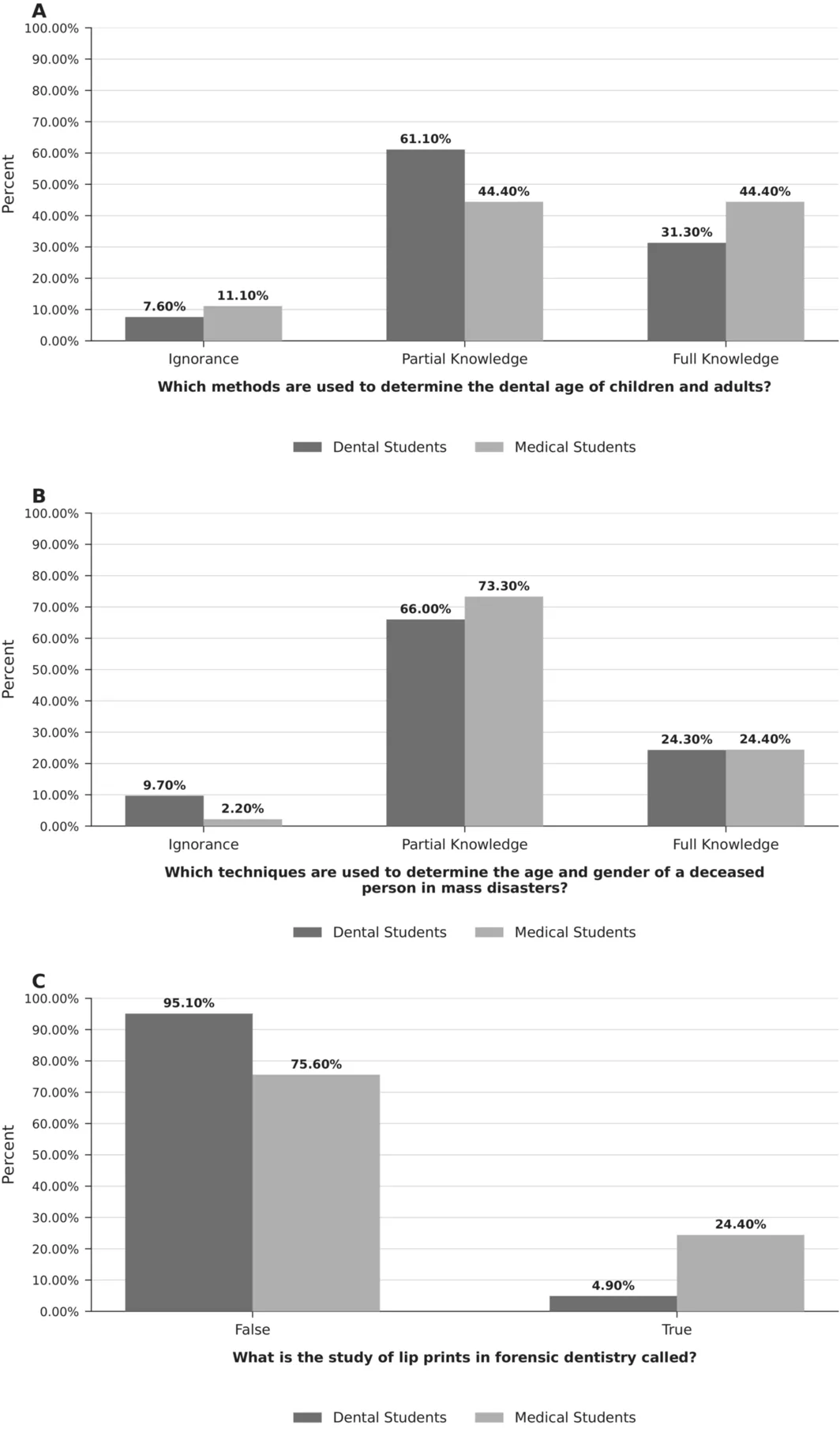
Bar graphs showing knowledge levels of dental and medical students on selected forensic odontology topics. (A) Methods used to determine the dental age of children and adults. (B) Techniques used to estimate age and gender in mass disasters. (C) Awareness of the term for lip print analysis in forensic dentistry. Knowledge was categorized as ignorance, partial or full in (A) and (B), and as correct or incorrect in (C). Bars indicate the percentage of students selecting each response.

## 4 Discussion

This study presents an exploratory descriptive assessment of forensic odontology awareness among dental and medical students in Turkey. Designed to identify educational trends the findings delineate patterns of exposure, contrasting specific areas of reported awareness with domains of limited conceptual familiarity. The primary findings indicate that while dental students reported higher frequencies of coursework and workshop attendance, their comprehension of core forensic concepts remained suboptimal. Critical domains, including bite mark analysis, the forensic application of dental DNA and cheiloscopy terminology, were correctly identified by a minority of respondents despite their curricular exposure. These results align with broader global trends. For instance, research in Cyprus indicated that while students recognize teeth as DNA repositories, few understand the legal responsibilities of the dentist as an expert witness [7]. Similarly, a multicentre study in India found that while record‐keeping was valued, only 18% of students knew the legal retention periods [8], a finding mirrored in Croatia where only 32% of dentists understood these mandates [18].

A notable finding was that medical students, despite less specialized curricular exposure, outperformed dental counterparts in domains such as lip print identification and DNA analysis. This disparity likely stems from fundamental differences in curricular architecture. Medical students receive consistent exposure to medicolegal principles through core disciplines such as pathology, legal medicine and medical ethics. This provides a broader foundation in human identification and legal documentation, even without specific forensic odontology training [19, 20]. Furthermore, medical education often integrates experiential learning, such as autopsy sessions and forensic simulations, which are reported by 92% of participants to enhance understanding of medicolegal processes [21].

Moreover, the emphasis on self‐directed learning and molecular life sciences in medical training supports a more robust comprehension of forensic topics like DNA profiling. In contrast, dental curricula have historically prioritized clinical and procedural skills with less emphasis on research involvement or critical literature appraisal [22]. These pedagogical distinctions may explain why medical students demonstrate superior performance in specific knowledge domains despite a lack of formal forensic odontology education.

The findings necessitate a shift from passive curricular exposure towards applied, interprofessional instructional models. We recommend incorporating structured simulation exercises, such as joint medical‐dental disaster victim identification (DVI) drills or simulated crime scene investigations. These experiential frameworks allow students to translate theoretical principles into medicolegal decision‐making while fostering professional accountability within multidisciplinary teams. Such approaches align with international standards prioritizing competency‐based and team‐oriented instruction in forensic education.

Radiology education provides a strategic entry point for applied training. Emphasizing systematic panoramic radiograph reporting that documents all restorative materials may enhance identification capacity during mass disasters. Teaching students to interpret images as both diagnostic and potential forensic records reinforces the dual clinical and legal significance of routine documentation. Similarly, foundational bite‐mark analysis can be integrated into preclinical prosthodontic training. Utilizing existing modules on occlusal morphology and dental anatomy to introduce bite‐mark principles may improve early recognition of forensic indicators, such as those found in cases of abuse without requiring substantial curricular expansion.

In this study, female students demonstrated higher levels of ethical awareness, a trend consistent with professional ethics literature. Prior research in dental education indicates that women often score higher on measures of moral sensitivity [7, 23, 24]. Carol Gilligan's care‐oriented moral framework provides a useful interpretive lens, describing a moral voice that prioritizes responsibility and responsiveness over strictly rule‐based justice‐oriented reasoning [25]. In forensic odontology, where ethical decisions involve victims' rights and the respectful handling of human remains, this care‐oriented framing may more closely align with assessed ethical dimensions. Evidence across health professions suggests these variations reflect differences in ethical framing rather than intrinsic capacity [26]. Consequently, forensic education should incorporate both justice‐oriented and care‐oriented frameworks to ensure a comprehensive approach to ethical reasoning.

The significance of these results is underscored by INTERPOL's DVI strategies, which categorize dental analysis as a primary identification method alongside dactyloscopy and DNA testing [27]. Evidence from resource‐limited settings, such as the Philippines following Typhoon Haiyan, illustrates the critical role of dental evidence when financial or cultural barriers restrict DNA analysis [5]. Despite the resilience of dental tissues, effective identification depends on the availability of accurate ante‐mortem records, which remains a systemic vulnerability in Turkey [3]. Our results show that only 13.9% of dental students recognized the forensic value of records, mirroring previous national findings where only 29.5% of dentists maintained documentation meeting forensic standards [13]. This deficiency often stems from clinical pressures where documentation is perceived as secondary to patient care. Adopting machine learning tools to automate the identification of restorations or impacted teeth from radiographs may reduce this administrative burden and enhance the forensic utility of routine archives [28, 29, 30, 31, 32].

Turkey's heavy reliance on DNA‐based identification indicates infrastructural gaps in dental data systems and broader systemic issues [33]. In mass fatality events, exclusive DNA use often suggests that dental data were either unavailable or that victims lacked prior access to formal care. Current data reveal that dental utilization in Turkey is largely symptom‐driven; over 60% of adults seek care only for pain, while only 25% attend routine check‐ups [34]. Therefore, infrastructure improvements must be coupled with public health initiatives promoting preventive visits and interdisciplinary awareness. Encouraging physicians to refer patients for dental evaluations could improve national readiness for large‐scale identification.

Finally, while 81.9% of dental students recognized signs of child abuse, only 9.0% identified the forensic relevance of bite marks. Among medical students, awareness was lower, with 41.1% identifying abuse indicators and 1.1% recognizing bite‐mark evidence. This mirrors international observations, where professionals demonstrate strong ethical intent but lack legal confidence and practical understanding of reporting protocols [35, 36]. The discrepancy between the intent to report and the knowledge of evidence highlights a critical gap in current education. Merely increasing exposure is insufficient; the depth and clinical context of forensic content must be improved to ensure that students can effectively navigate the legal and forensic infrastructure of their professions [37].

These findings should be interpreted within the context of several methodological constraints. First, although the survey instrument underwent expert review for content validity and pilot testing for face validity, it was not subjected to formal psychometric scaling. As the instrument functions as a broad knowledge index covering heterogeneous domains (e.g., legal protocols vs. anatomical techniques) rather than a scale measuring a single latent construct, internal consistency metrics such as Cronbach's alpha were not applicable.

Additionally, the cross‐sectional nature of the study precludes causal inferences regarding the direct impact of specific curricular components. Potential unmeasured confounders, such as individual student interest or independent study habits outside the formal curriculum, were not controlled.

The study is also subject to various forms of bias. Social desirability bias likely influenced ethically sensitive items, such as child abuse reporting, where responses may reflect perceived professional expectations rather than actual preparedness. Evidence of overestimation bias was observed in the discrepancy between high self‐reported sufficiency and low objective accuracy on technical items, such as bite‐mark awareness. Furthermore, the mandatory nature of the survey items, while strictly ensuring data completeness, may have inadvertently prompted satisficing behaviour among participants experiencing cognitive fatigue.

Regarding the sampling strategy, the use of a non‐probability convenience approach via institutional channels introduces potential selection bias, as students with a preexisting interest in forensics may have been more inclined to participate. As emphasized in the CHERRIES framework, the architectural limitations of the survey platform prevented the quantification of a precise denominator or the tracking of unique site visitors. Consequently, standard metrics such as view, participation and completion rates could not be calculated [15]. Structural and sampling factors limit the external validity and cross‐study comparability of the findings. These results should be viewed as reflective of general educational patterns rather than a validated measure of forensic competency.

Global discourse reflects a growing consensus that forensic odontology is foundational to the ethical and scientific responsibilities of healthcare professionals. While Turkey's national dental curriculum (DUCEP‐2024) serves as a responsive framework, it currently addresses forensic topics implicitly. Mid‐level competencies, such as medical report preparation, are established, yet a dedicated framework for human identification, age estimation and disaster protocols remains largely undefined. This gap offers an opportunity for strategic development informed by international standards, such as those of the American Board of Forensic Odontology (ABFO) and Model Curriculum and the Organization of Scientific Area Committees (OSAC), which advocate for technical proficiency paired with legal literacy. Due to the absence of a mandatory national core curriculum for forensic odontology in Turkey, the ‘formal coursework’ reported by participants likely represents a heterogeneous mix of elective courses, brief seminar series and varying depth of content. Consequently, we could not isolate the specific impact of curricular duration or syllabus composition on student knowledge. While our findings demonstrate that some level of exposure correlates with higher awareness, future research should prioritize a comprehensive curricular review to determine the optimal pedagogical standards specifically, the credit load and practical components required to achieve professional competency. Addressing the challenges of curricular integration requires interdisciplinary collaboration between forensic specialists, medical‐legal professionals and pathology departments. Integrating forensic odontology into existing pathology, ethics and interprofessional modules will significantly enhance student preparedness for medico‐legal responsibilities. Ultimately, embedding these principles into undergraduate education represents a strategic investment in public health and legal accountability, ensuring that graduates are equipped to serve at the intersection of human identification, legal truth and societal safety.

## 5 Conclusion

This study identifies substantial disparities in forensic odontology knowledge between dental and medical students in Turkey, with varying degrees of curricular exposure. The findings reveal a distinct discrepancy between reported educational exposure and objective knowledge. Although dental students participated in formal coursework and workshops at higher rates, this exposure was not uniformly associated with higher recognition of core forensic concepts compared to medical students. Furthermore, substantial knowledge deficits were observed across both cohorts regarding the utility of dental records, disaster victim identification and legal documentation of abuse. These results indicate that under the current educational framework, the quantity of coursework does not automatically correlate with the theoretical proficiency required for medicolegal responsibilities. The observed disconnect between participants' self‐rated sufficiency and their objective performance suggests that the current mode of educational delivery may not be fully meeting the learning needs of future practitioners.

## Author Contributions

E.N.K.: conceptualization, methodology, investigation, formal analysis, data curation, writing – original draft, visualization, project administration. B.Y.: supervision, writing – review and editing, validation.

## Funding

The authors have nothing to report.

## Ethics Statement

The study protocol was approved by the Istinye University Human Research Ethics Committee (Approval: 24–272). Participation was voluntary. To maintain data integrity, the survey platform was configured to restrict submissions to a single entry per user. Although this required a temporary login for validation, the email collection feature was disabled to ensure anonymity. Electronic informed consent was obtained via the opening interface, which clarified that the login process was for validation only and that no personal identifiers were recorded.

## Conflicts of Interest

The authors declare no conflicts of interest.

## Supporting information


**File S1:** The Forensic Odontology Awareness and Knowledge Questionnaire utilized in this study, which assesses participants' demographics, self‐reported familiarity with forensic concepts, specific knowledge of forensic techniques and awareness of child abuse indicators.

## Data Availability

The data that support the findings of this study are available from the corresponding author upon reasonable request.
