# Supplementary material for: Comparative Assessment of Forensic Odontology Awareness Among Dental and Medical Students in Turkey
Source: Eur J Dent Educ. 2026 Mar 28;30(4):1951–63. doi: 10.1111/eje.70148 (PMC13686663; doi:10.1111/eje.70148)
Supplement: Supplementary file 1 [file EJE-30-1951-s001.docx]

**Forensic Odontology Awareness and Knowledge Questionnaire**

*What is your gender?*

- Male
- Female
- Other (please specify): ______
- Prefer not to say

*Which of the following best describes your age?*

- 18–21 years
- 22–25 years
- Over 25 years

*What is your current academic year and program?*

- 4th year (Dentistry)
- 5th year (Dentistry)
- 5th year (Medicine)
- 6th year (Medicine)

1. **Have you completed any formal coursework specifically covering forensic odontology?**

- Yes
- No

2. **Have you attended any workshops, seminars, or trainings focused on forensic odontology within the past two years?**

- Yes
- No

3. **What is the source of your knowledge about forensic dentistry?**
   (You may select more than one)

- Books
- Internet
- Workshops
- Seminars
- Lectures

4. **Are you familiar with the concept of forensic odontology as a branch of dentistry?**

- Yes
- No

5. **Are you familiar with the concept of forensic odontology as a branch of forensic medicine?**

- Yes
- No

6. **Are you familiar with the concept that dentists may testify as expert witnesses in court for forensic cases?**

- Yes
- No

7. **Are you familiar with the concept that maintaining accurate dental records contributes to quality patient care?**

- Yes
- No

8. **Are you familiar with the concept that maintaining accurate dental records can assist in forensic identification?**

- Yes
- No

9. **Are you familiar with the concept of estimating biological sex using teeth in forensic cases?**

- Yes
- No

10. **Are you familiar with the concept of age estimation through forensic odontology?**

- Yes
- No

11. **Are you familiar with the concept of using teeth as a source of DNA in forensic investigations?**

- Yes
- No

12. **Are you familiar with the concept of bite mark analysis in forensic investigations?**

- Yes
- No

13. **Are you familiar with the concept that forensic odontology is used to help identify deceased individuals or crime suspects?**

- Yes
- No

14. **Which methods are used to determine the dental age of children and adults?**

- Eruption patterns
- Histological methods
- Biochemical methods
- I do not know

15. **Which techniques are used to determine the age and sex of a deceased person in mass disasters?**

- Reconstruct body
- Dental records
- Fingerprints
- I do not know

16. **What is the study of lip prints in forensic dentistry called?**

- Lipology
- Cheiloscopy
- Dermatoglyphics
- I do not know

17. **Would you describe your knowledge and awareness of forensic odontology as sufficient?**

- Yes
- No

18. **Are you interested in participating in workshops and seminars in forensic odontology?**

- Yes
- No

19. **Which of the following signs may indicate child abuse?**

- Physical injuries
- Behavioral changes
- Clothing
- Any scars
- All the above
- I do not know

20. **What would you do if you suspected a child was being abused?**

- Inform police
- Inform parents
- Take no action

Note: The items in this questionnaire were adapted from previously published studies [1, 2].

**References**

1. Giannakopoulos K, Lambrou-Christodoulou P, Kaklamanos EG. Awareness of forensic odontology among dental students and faculty in Cyprus: a survey-based study. Dent J (Basel) 2024;12(1):6. doi:10.3390/dj12010006

2. Kumaraswamy J, Nagarajachar RB, Keshavaiah R, Susainathan A, Sreenivas Reddy MB, Naidu J. A cross-sectional study to assess knowledge, attitude, and awareness of forensic odontology among medical students: an emergency concern. Int J Forensic Odontol 2018;3(1):17-20. doi:10.4103/ijfo.ijfo_3_18
